# Bisphenol A removal from aqueous solutions using novel UV/persulfate/H_2_O_2_/Cu system: optimization and modelling with central composite design and response surface methodology

**DOI:** 10.1186/s40201-016-0255-x

**Published:** 2016-12-01

**Authors:** S. Ahmad Mokhtari, Mehdi Farzadkia, Ali Esrafili, Roshanak Rezaei Kalantari, Ahmad Jonidi Jafari, Majid Kermani, Mitra Gholami

**Affiliations:** 1 Research Center for Environmental Health Technology, School of Public Health, Iran University of Medical Sciences, Tehran, Iran; 2 Department of Environmental Health Engineering, School of Public Health, Iran University of Medical Sciences, Tehran, Iran; 3 Department of Environmental Health Engineering, School of Public Health, Ardabil University of Medical Sciences, Ardabil, Iran

**Keywords:** BPA, UV/SPS/HP/Cu, Aqueous solutions, CCD

## Abstract

**Background:**

Bisphenol A is a high production volume chemical widely used in manufacturing polycarbonate plastics and epoxy resins used in many industries. Due to its adverse effects on human health as an endocrine disruptor and many other effects on the various organs of the human body as well as aquatic organisms, it should be removed from the aquatic environments. This study aimed to mineralisation of BPA from aquatic environments by application of novel UV/SPS/H_2_O_2_/Cu system and optimization and modelling of its removal using central composite design (CCD) from response surface methodology (RSM).

**Methods:**

CCD from RSM was used for modeling and optimization of operation parameters on the BPA degradation using UV/SPS/HP/Cu system. Effective operation parameters were initial persulfate, H_2_O_2_, Cu^2+^ and BPA concentration along with pH and reaction time, all in three levels were investigated. For analysis of obtained data ANOVA test was used.

**Results:**

The results showed that a quadratic model is suitable to fit the experimental data (*p* < 0.0001). Analysis of response surface plots showed a considerable impact of all six selected variables which BPA and Cu^2+^ initial concentrations have been the highest and the least impact on the process, respectively. *F*-value of model was 54.74 that indicate significance of the model. The optimum values of the operation parameters were determined. The maximum removal of BPA was achieved 99.99 % in optimal conditions and in that condition TOC removal was about 70 %. Finally, validation and accuracy of the model were also evaluated by graphical residual analysis and the influential diagnostics plots. The higher relevance between actual and predicted values demonstrated the validation and applicability of the obtained equation as the model.

**Conclusions:**

According to the results, UV/SPS/HP/Cu system is an effective process in degradation and mineralisation of BPA and CCD methodology is a convenient and reliable statistical tool for optimizing BPA removal from aqueous solutions.

## Background

During the last decade, the so-called emerging contaminants (CECs or ECs) have assigned the majority of environmental researches. This is due to concerns about the potential effects of these chemicals on human health and the environment [1–4]. A group of these pollutants is endocrine disrupting chemicals (EDCs) and potential EDCs are mostly man-made, found in various materials such as pesticides, metals, additives or contaminants in food, and personal care products. BPA (CAS No: 80-05-7), one of the most important EDCs, has a solid state with white colour which often used as an intermediate in the production of epoxy, polycarbonate, polysulfone and resins of special polyester. In recent centuries because of BPA presence in variety of products such as packaging of beverage and food, adhesives, construction materials, retardants, electronic equipment, and paper coatings [5, 6]. It has shown that BPA has hormone-like properties at higher concentrations, so causes an increase in concerns about its entrance to the environment as well as its suitability in different applications such as consumption goods and packaging for some food products [3].

Several studies have been shown BPA’s side and adverse health effects on human and animals. Human contact with BPA can occurs often through ingestion of contaminated food and water, dust from inhaling gases and particulates in the air, and also via the skin [7, 8]. BPA is a nonsteroidal xenoestrogen which shows about 10–4 times higher than estradiol activation [9]. Another work indicates that BPA may be has an equivalent effect of estradiol in triggering some responses of receptors [10] and probably it act as antagonist for androgen receptor [11, 12]. Other important health issues related to BPA that reported in the literature are high rates in the incidence of diabetes, breast and prostate cancers, and decreased sperm count, sexual problems, premature puberty, obesity, nerve problems and etc. [13]. According to the EPA and FDA, intake limits for BPA in human health assessment was determined 0.05 mg/kg.d and 0.005 – 0.5 mg/kg.d respectively; also European Union has determined 1.5 μg/L as PNEC^1^ for aquatic organisms. Based on current information all toys have plastic in their structure must not contain BPA.

BPA can enter wastewater treatment plants through both discharge directly to the sewage system and surface runoff, and in the next steps it has been entered in influent and effluent also in residual sludge in wastewater treatment plants (WWTPs) [14]. In fact, WWTPs are considered to be the main secondary sources of BPA pollution due to their imperfect and defective performance of treatment process [15–17].

It has been demonstrated that several processes including ultrafiltration, RO, NF, photo-catalysis and membrane filtration, hollow fibre microfiltration membrane, single walled carbon nanotubes–ultrafiltration, coagulation and adsorption, laccase catalyzation and atc are capable of removing BPA from aqueous environments with different removal efficiencies [18–27].

Newly, another oxidation method was introduced based on PS that has high redox potential (E_0_ = 2.01 V), and it is applied less frequently compared to ozone (E_0_ = 2.07 V); The dissociation of PS in the aquatic environment is a major stage for yielding sulphate radicals (SO_4_^−•^, E_0_ = 2.60 V), for strong oxidation, and these radicals also react with water for generating hydroxyl radicals that both of generated radicals can react with organic matter and degrade it to the products [15, 28–32]:

1 $$ {\mathrm{S}}_2{{\mathrm{O}}_8}^{\hbox{-} 2} + \mathrm{initiator}\to \mathrm{S}{{\mathrm{O}}_4}^{\bullet \hbox{-} } + \left(\mathrm{S}{{\mathrm{O}}_4}^{\hbox{-} \bullet}\mathrm{or}\ \mathrm{S}{{\mathrm{O}}_4}^{\hbox{-} 2}\right) $$

2 $$ \mathrm{S}{\mathrm{O}}_4{{}^{\hbox{-}}}^{\bullet } + {\mathrm{H}}_2\mathrm{O}\ \to\ {\mathrm{H}}^{+} + \mathrm{S}{{\mathrm{O}}_4}^{2\hbox{-} } + \mathrm{H}{\mathrm{O}}^{\bullet } $$

Oxidation of organic matters with PS has some advantages than other oxidizing agents due of its convenient storage and transportation, being in solid state at the normal temperatures, high ability of water solubility, relatively lower costs, and high stability; another advantage of AOPs (as well as sulphate base radicals) is degradation and mineralization of recalcitrant and/or toxic organic pollutants and lack of leaving residues and sludge [33–36].

There are few methods for activation of PS ion, initiation, and then sulphate radical production, which are included conventional methods (*heating, catalysis with transition metal, and UV irradiation*) and new methods (*photochemical, iron and chelated iron ion, zero-valent iron, minerals, activated carbon, microwave, and integrated* activation) [33].

In this study, it was used an integrated method for degradation and mineralization of BPA which consist of UV, SPS (Sodium persulfate), H_2_O_2_, and Cu cation. This study was conducted with the propose of evaluating BPA removal efficiency by UV/SPS/HP/Cu system and survey the effect of factors, including BPA initial concentration, time, pH, PS, peroxide and Cu ion concentrations and finally optimization of BPA removal with their factors by CCD.

Generally, treatment processes of pollution are optimised using “*one factor at the time*”, such as most of other processes in industries with variating of its variables. Moreover, this method assumes that various treatment parameters do not interact and that the response variable is only function of the single varied parameter. In addition, the response obtained from pollution treatment procedure, for instance finding from the interactive effects of the various variables. When a mix of multiple independent variables and interactions of them affect given responses, response surface methodology (RSM) is a proper and efficient tool in optimization of the response [37]. In RSM an experimental design is used like CCD for a fitting model by minimum squares technique [38, 39]. In the next stage, adequacy of the obtained model is demonstrated by using the diagnostic tests presented by analysis of variance (*ANOVA*). The plots of response surface can be applied to survey the surfaces and location of the optimized point. In most process of industrials, RSM is normally applied for evaluation of the results and efficacy of the operations [40, 41].

In present study, a CCD in the form of full factorial was applied for developing mathematic relations, in terms percentage of removal, providing quantitative aassessment of the UV/SPS/HP/Cu system used to treat BPA.

## Methods

### Chemicals and samples

The model contaminant, BPA ≥ 99 %, was purchased from Aldrich (SKU-Pack Size). Analytical grade n-hexane and acetone, methanol were all provide by Merck (Darmstdat, Germany). Other chemicals such as SPS, HP, copper sulphate (Cu_2_SO_4_), sulphuric acid (H_2_SO_4_), Sodium hydroxide (NaOH), monosodium phosphate and etc. also purchased from Merck. Deionized water was supplied by a home-made deionizer. Stock standard solutions of BPA (10,000 mg/L) were prepared in ethanol and were then diluted with water (as working solutions) and stored at −18 ± 2 °C. The calibration curve was constructed of six calibration standard solutions (1–50 mg/L; CS) were prepared by adding the diluted working solution into the deionized water samples. The validation tests were carried out using two quality controls (QC, 2 and 5 mg/L). Both CS and QC samples were stored at −18 ± 2 °C until analysis. Taken samples from system at the specified times, immediately was injected to the HPLC; If necessary, to stop the reaction, methanol was added to the samples.

### Instrumentation

The photochemical-degradation studies were carried out in a batch reactor system. The reactor used in this study, the tubular steel, was made from stainless steel, which was available for the high reflection of the radiation, resistant to chemical and cylindrical shape, was designed and built with a diameter about 8 cm and a length of 90 cm. Irradiation was achieved by a low-pressure mercury lamp (Philips, TUV 30 W/GC T8) with UV radiation 12 W located axially in the centre of reactor and was placed inside the quartz tube. The light length of the lamp and quartz sleeve diameter of 30 mm was same with reactor that emits approximately 90 % of its radiation at 253.7 nm. The chemicals used in each run along with aqueous solution with a volume of 2 l was added in the reactor, which was placed between the reactor walls and UV lamp system. To make a uniform solution a shaker was used with 100 rpm.

The samples containing BPA, were prepared from the stock solution, and their residual concentrations in the samples were analysed by HPLC (Cecil CE 4100) using a Hypersil C18 column (250 mm × 4.6 mm i.d, with 5 μm particle size) with a UV detector (Cecil CE 4200) at 230 nm. The Mobile phase consisted of a mixture of methanol and deionized water with a ratio of 0.8:0.2 (v:v) at a flow rate of 1 mL/min. 20 μL of sample was injected using a Hamilton syringe to the injector. The detection limit of method for BPA was 1 μg/L.

Other equipment used in this study were TOC analyser (analytikjena, multi N/C 3100), GC-MS (Agilent Technologies 7890A, USA), pH meter (HQ11D, Portable), distillation apparatus (GFL, Germany), spectrophotometer (DR 6000 UV/VIS, HACH, USA), laboratory precision scale (BEL Engineering, Italy) and variety of laboratory glass containers and accessories.

### Experimental design and data analysis

A CCD in the form of full factorial design was used, in which six independent variables were converted to dimensionless ones (*x*_*1*_ *, x*_*2*_ *, x*_*3*_ *, x*_*4*_ *, x*_*5*_ *, x*_*6*_), with the coded values at 3 levels: −1, 0, +1 (Table 1). Preliminary optimal variable levels were obtained using multi-simplex method. The arrangement of CCD in mentioned 86 runs, was in such a way that allows the development of the appropriate empirical equations (second order polynomial multiple regression equations):

**Table 1:** Independent variables and their levels for the central composite design used in this study

Variable	Unite	Symbol	Coded variable levels		
			−1	0	1
C persulfate	*mg/L*	*x*_*1*_	25	65	105
C peroxide	*mg/L*	*x*_*2*_	5	10	15
pH	*-*	*x*_*3*_	3	7	11
Cu^+^	*mg/L*	*x*_*4*_	5	17.5	30
Time	*min*	*x*_*5*_	5	122.5	240
C BPA	*mg/L*	*x*_*6*_	5	22.5	40

3 $$ y={\beta}_0+{\beta}_1{x}_1+\cdots +{\beta}_6{x}_6+{\beta}_{11}{x}_1^2+\cdots +{\beta}_{66}{x}_6^2+{\beta}_{12}{x}_1{x}_2+\cdots +{\beta}_{56}{x}_5{x}_6 $$

And therefore, the predicted response (y, or BPA removal percentage) was correlated to the set of regression coefficients (β): the intercept (β_0_), linear (β_1_, β_2_, β_3_), interaction (β_12_, β_13_, β_23_) and quadratic coefficients (β_11_, β_22_, β_33_).

The “Design expert” (version 7), SPSS (version 18), and excel 2013 softwares were used for regression and graphical analyses of the obtained data.

### Toxicity test

To evaluate the acute toxicity of effluent containing BPA and its intermediate products before and after treatment and degradation by UV/SPS/HP/Cu system, bioassay test with Daphnia Magna, as an indicator, was done as detailed in standard methods for the examination of water and wastewater. Exposure times 12, 24, 48, 72, and 96 h was considered, and the number of immobilized and dead infants was recorded and then LC_50_ values were calculated by usage of PROBIT regression in the SPSS software (version 23) and the toxic unit (TU) of effluent was determined by TU = 100/LC_50_. Also for identify intermediate products and their possible role in creation of toxicity, GC-MS analysis was carried out.

## Results and discussion

### Selecting the ratio for reagents

The overall efficiency of the UV/SPS/HP/Cu system was determined with multi-simplex method as a results of our pre-test experiments (Results not shown). Based of obtained results from this work and similar work, it was found PS ion, hydrogen peroxide, Cu^2+^ ion, pH, reaction time and initial BPA concentration all are key parameters and contribute to the proper performance of system and its removal efficiency depends on them and the their relationships in terms of formation and utilization of sulphate and hydroxyl radicals [15, 42]. In the present study, with the aim of reducing the use of chemicals and their related cost saving, and take advantage of potential synergistic property, chemicals and other applied parameters such as UV irradiation applied simultaneously. Initially different levels of chemicals and parameters were selected and applied alone in the wider ranges for each parameter (UV lamp: 30 Wat as fixed; SPS: 0.1–10 as molar ratio; HP (Hydrogen peroxide): 0.1–5 as molar ratio; Cu^2+^: 0.1–2; BPA: 5–40 mg/L); in this stage it was shown that use of the each variable alone, have the less efficiency in removal and degradation of BPA and for more effectiveness PS must be active with activation factors such as UV, Cu^2+^ and hydrogen peroxide [43–45]. Excess amounts of hydrogen peroxide and PS is not recommended since unutilized hydrogen peroxide contributes to COD (1 mg/L of H_2_O_2_ contributes 0.25–0.27 mg/L to the COD concentration), and the remaining of PS can be exhibit as contaminant in the effluent [46, 47]. Also, PS and H_2_O_2_ in large quantity act as radical scavenger and lead to duplicate of reagent consumption for activation of PS and therefore probably reducing removal efficiency of system [48, 49]. Moreover, used ratios and concentrations applied in the present study were in the range reported by others when the AOPs based sulphate and hydroxyl radicals have been applied to the removal and degradation of BPA in the water and wastewater [15, 44, 50]. According to results, some levels and ratios of variables was excluded and the following levels were chosen: SPS in ranges 0.5–2, HP in ranges 0.5–2, Cu^2+^ in ranges 0.1–1 as molar ratio to BPA, and finally BPA in range 5–40 mg/L.

### CCD and fitted regression models as related to the BPA removal

In this work, the relationship between one criteria of the pollutant removal (named BPA removal) and six controllable factors (namely SPS, HP and Cu^2+^ initial concentrations, pH, reaction time, and BPA initial concentration) was studied. CCD with 86 runs allows us the development of mathematical equation where response variable y (BPA removal) is assessed as a function of SPS concentration (*x*_*1*_), HP concentration (*x*_*2*_), Cu^2+^ concentration (*x*_*3*_), pH (*x*_*4*_), reaction time (*x*_*5*_) and BPA initial concentration (*x*_*6*_) and calculated as the sum of a constant number, six first-order effects (terms in *x*_*1*_, *x*_*2*_, … and *x*_*6*_), thirteen interaction effects (terms in *x*_*1*_ *x*_*2*_, *x*_*1*_ *x*_*3*_, …. and *x*_*5*_ *x*_*6*_) and six second-order effects (*x*_*12*_, *x*_*22*_, … and *x*_*62*_) according to the Eq. (4).

4 $$ \begin{array}{l}\boldsymbol{Y}\ \left({}_{\boldsymbol{BPA}\ \boldsymbol{Removal}}\right) = 84.75 + 2.18\ {x}_1 + 1.74\ {x}_2 + 2.64\ {x}_3 + 0.9\ {x}_4 + 4.36\ {x}_5 + 4.52\ {x}_6\hbox{--}\ 0.12\ {x}_1{x}_2\hbox{--} \\ {}\ 0.04\ {x}_1{x}_3\hbox{--}\ 0.083\ {x}_1{x}_4\hbox{--}\ 0.20\ {x}_1{x}_5 + 0.13\ {x}_1{x}_6\hbox{--}\ 0.042\ {x}_2{x}_3 + 0.082\ {x}_2{x}_5 + 0.074\ {x}_2{x}_6 + 0.23\ {x}_3{x}_5\\ {} + 0.41\ {x}_3{x}_6 + 0.45\ {x}_4{x}_5 + 0.13\ {x}_4{x}_6 + 0.039\ {x}_5{x}_6\hbox{--}\ 4.30\ {x_1}^2\hbox{--}\ 2.5\ {x_2}^2 + 7.10\ {x_3}^2\hbox{--}\ 3.85\ {x_4}^2\mathit{\hbox{-}}\ 3.5\ {x_5}^2\\ {} + 0.3\ {x_6}^2\end{array} $$

By removing the model terms are not significant statistically, obtained model is reduced to the following form that can be improved it.

5 $$ \begin{array}{l}\boldsymbol{Y}\ \left({}_{\boldsymbol{BPA}\ \boldsymbol{Removal}}\right) = 84.76+2.18\ {x}_1+1.74\ {x}_2+2.64{x}_3+0.9{x}_4 + 4.36\ {x}_5 + 4.52\ {x}_6 + 0.45\ {x}_4{x}_5+\\ {}4.30\ {x_1}^2\hbox{--}\ 2.5\ {x_2}^2 + 7.10\ {x_3}^2\hbox{--}\ 3.85\ {x_4}^2\hbox{-}\ 3.5\ {x_5}^2\end{array} $$

Then in the next stage, to evaluation of fitness for achieved model, the obtained results were analyzed using ANOVA (analysis of variance). The model for BPA removal (Y) was considerable significant by using F-test with confidence level 5 % (0.05 > F < Prob). Obtained *F*-value of 54.74 for model indicate its significance. There is just 0.01 % possibility that a “Model F-Value” could cause by the error. Equation 4 as the fitted regression model (in terms of coded values for the variables) were applied to quantitative analysis of the impact of SPS, HP, and Cu^2+^ concentration, pH, time and initial BPA concentration on the description of UV/SPS/HP/Cu system for the BPA removal.

The statistical parameters of the ANOVA for the model of BPA removal are provided in Table 2. Since R^2^ always reduces whenever a variable is dropped from a regression model, in statistical modelling the adjusted R^2^ which takes the number of regressor variables into account, is usually selected [51]. The R^2^ coefficient gives the proportion of the total variation in the response variable explained or accounted for by the predictors (*x*’s) included in the model [52]. In this work, the adjusted and predicted R^2^ were calculated to 0.9447 and 0.9215, respectively. Adequate precision index for the model was obtained 32.885; this index measures “signal to noise ratio” in the model, which statistically ratio that higher than four is desirable [53] and obtained ratio for the model is much higher than this (32.85). The R^2^ factor in this work certified a suitable match of the quadratic model to the experimental data.

**Table 2:** Statistical parameters obtained from the ANOVA for the model

R^2^ (R squared)	0.9622
R^2^ adjusted	0.9447
Predicted R-Squared	0.9215
Prob > F	<0.0001
Std. Dev.	1.74
C.V. %	2.19
PRESS	365.41
Adequate precision	32.885

As it has been provided in Table 2, the C.V. for model has been calculated to be %2.19. The C.V. as the ratio of standard error of estimate for the mean value of the observed response (as a percentage) is a measure of reproducibility of the model and as a general rule can be considered reasonably reproducible if its CV is not greater than 10 % [37, 53]. The model fitted for BPA removal had a relatively good CV. This model had high R^2^ value and showed no lack of fit (Lack of fit *F*-value = 0.49). Diagnostic plots were applied for further evaluation of model such as Normal probability plot of the studentized residuals (to check for normality of residuals), Studentized residuals versus predicted values (to check for constant error), Externally Studentized Residuals (to look for outliers, i.e., influential values), and Box-Cox plot (for power transformations); and the assumptions of normality, independence and randomness of the residuals were satisfied. Using Box-Cox plot, Lambda value was obtained equal 1.0, with range Low C.I. = −2.24 and High C.I. = 1.15. Thus the predicted model can be used to navigate the space defined by the CCD.

The relative contribution of each factor to dependent variable (y: BPA removal) was directly measured by the respective coefficient in the fitted model. A positive sign for the coefficients (β) in the fitted model for Y indicated that the level of pollutant removal increased with increased levels of factors from *x*_*1*_ to *x*_*6*_. A review of these coefficients shows that related coefficient with *x*_*6*_ factor (BPA initial concentration) has the highest value and therefor the highest effect and obtained model has the most sensitivity for *x*_*6*_ with the equivalent 4.52; And other factors, including *x*_*5*_, *x*_*3*_, *x*_*1*_, *x*_*2*_, and *x*_*4*_ have been allocated the next ranks in sensitivity of the model for them. On the other hand, these coefficient’s ranks and arrangement of them in the model indicate that the removal of BPA as a model pollutant is affected by these factors by the mentioned order. Moreover, the existence of a negative sign for the regression coefficient of some factors, indicates that the ability of the system decreases with that factor’s value in the removing of the BPA. Of course, this issue is true just about some coefficients related to interactions and quadratic form of factors. In order to better understanding of the obtained results, the predicted model is provided in Figs. 2, 3, 4, 5 and 6 as the three-dimensional response surface plots. However, to validate the model, 15 separate experiments with different condition of applied parameters was done. The predicted values for BPA removal in this study are given in Table 3, which includes the measured data for these response variables. Comparing experimental values with the values predicted by the mathematical model indicate 92.56 % correlation between them, that which represents adequacy and suitability of model in predicting of the responses.

**Table 3:** Experimental and theoretically predicted values for BPA removal based on obtained model

Experiment no.	Actual value	Predicted value
1	91.59	88.85
2	71.47	73.30
3	75.19	76.50
4	89.21	87.10
5	83.22	81.60
6	72.88	75.95
7	82.35	87.60
8	81.50	78.90
9	90.91	88.65
10	81.14	82.95
11	81.20	85.15
12	85.26	84.10
13	77.83	76.95
14	92.27	97.55
15	85.17	90.74

### Optimization of BPA removal and response surface plotting

**Fig. 1:**
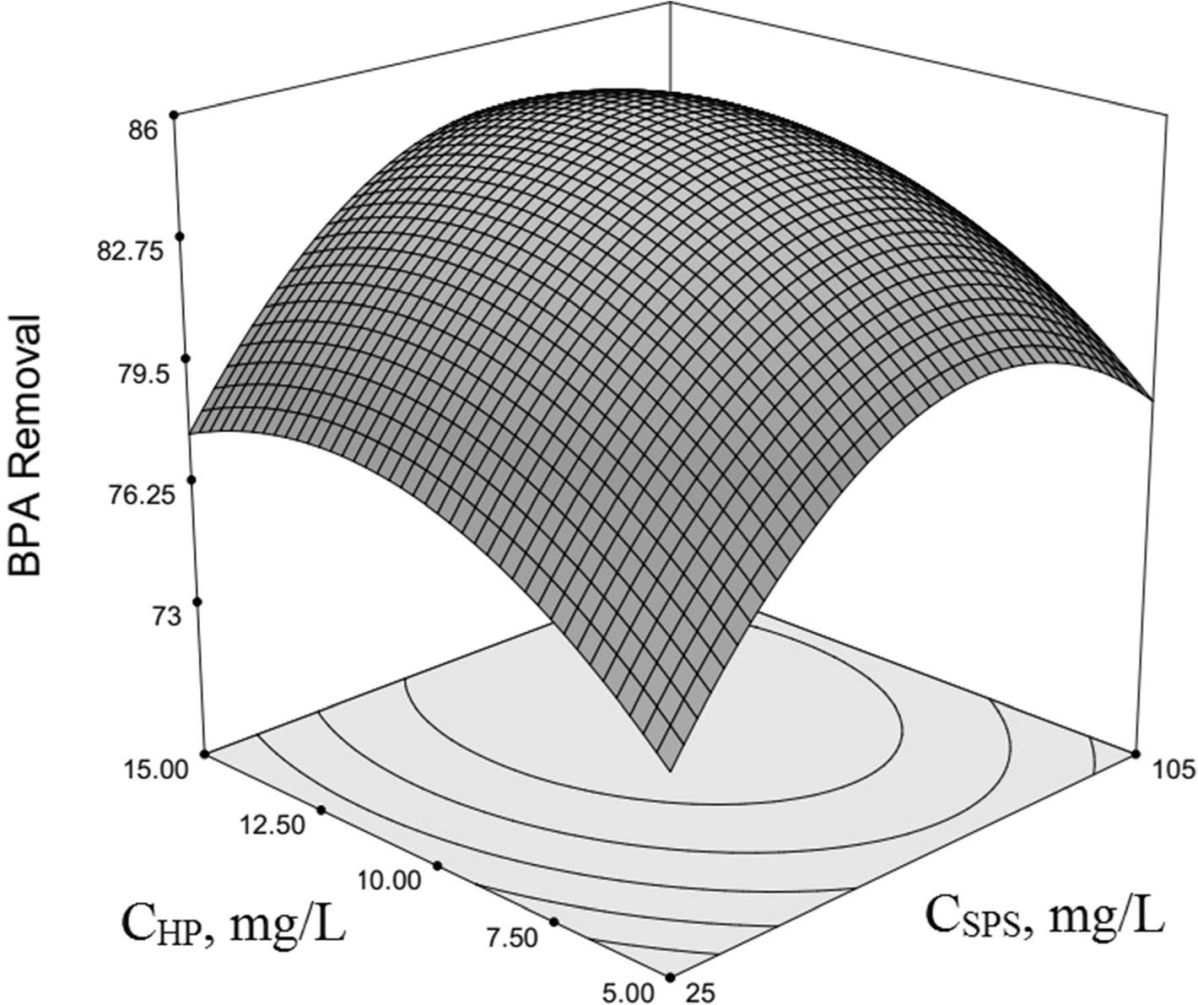
Second-order response surface plot in the BPA removal for the UV/SPS/HP/Cu system. Dependence of y on the SPS and HP concentrations. (pH = 7, Cu^2+^ = 17.5 mg/L, Time = 122.5 min, BPA = 22.5 mg/L)

**Fig. 2:**
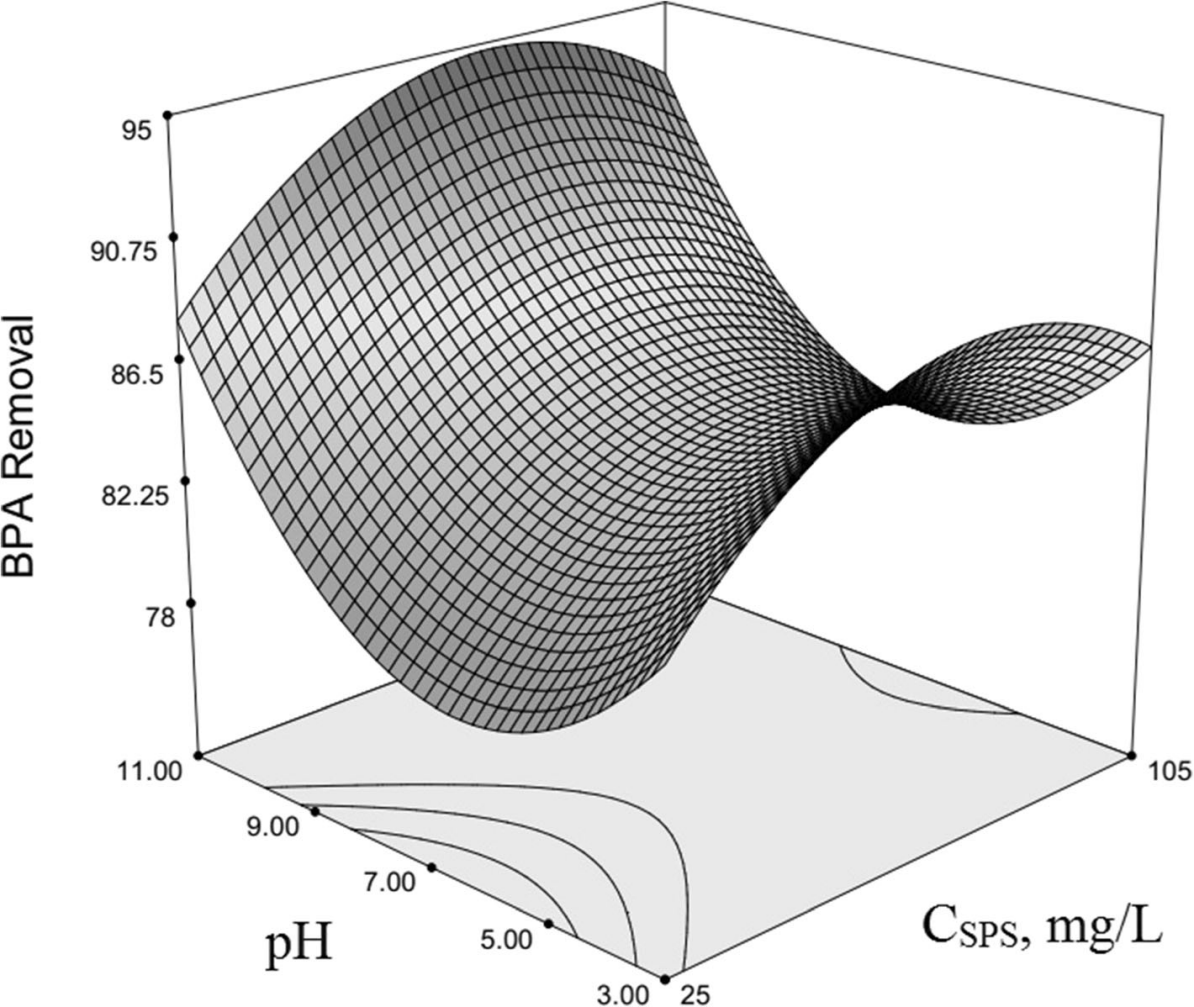
Second-order response surface plot in the BPA removal for the UV/SPS/HP/Cu system. Dependence of y on the SPS and pH concentrations. (HP = 10 mg/L, Cu^2+^ = 17.5 mg/L, Time = 122.5 min, BPA = 22.5 mg/L)

**Fig. 3:**
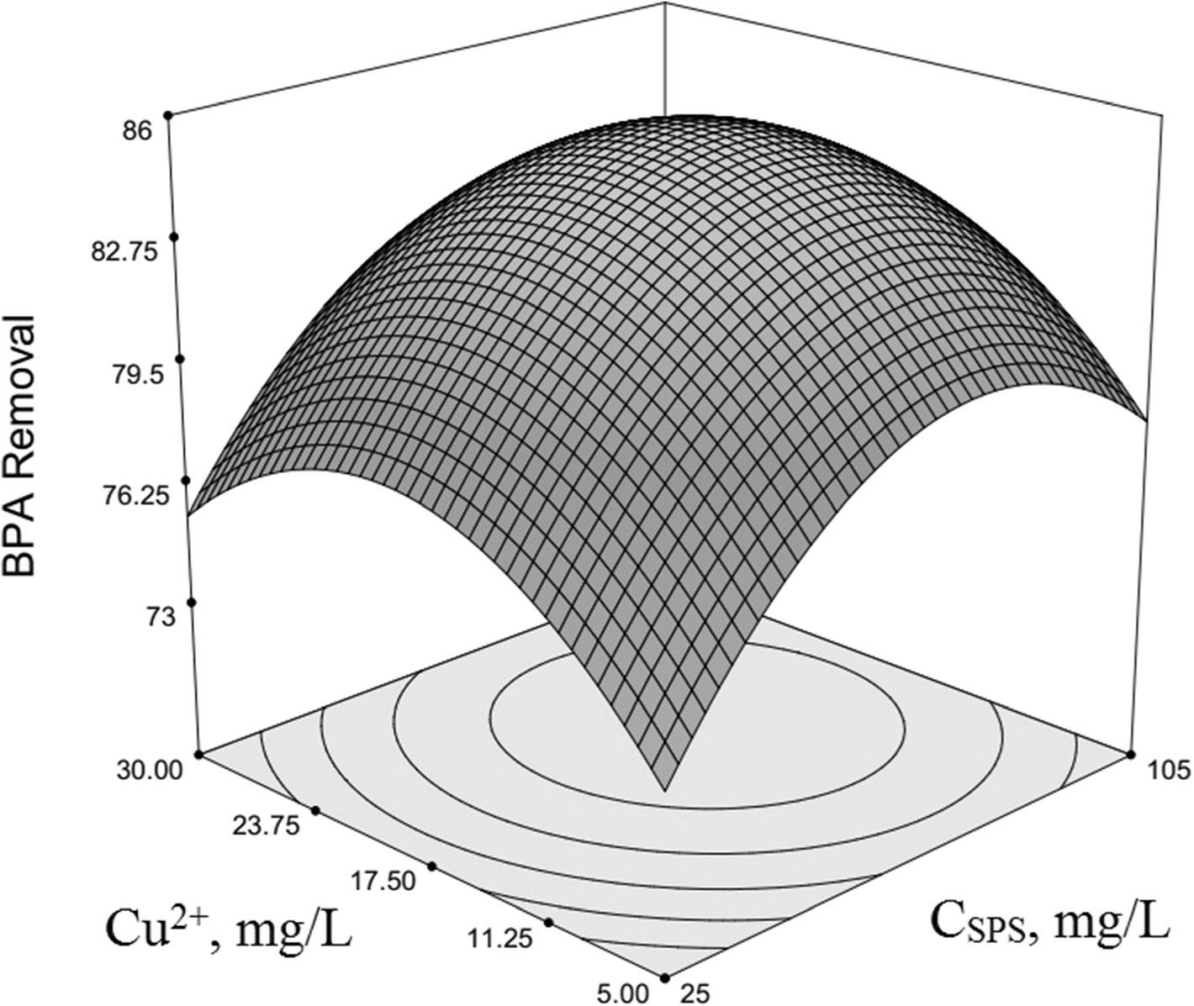
Second-order response surface plot in the BPA removal for the UV/SPS/HP/Cu system. Dependence of y on the SPS and Cu^2+^ concentrations. (HP = 10 mg/L, pH = 7, Time = 122.5 min, BPA = 22.5 mg/L)

**Fig. 4:**
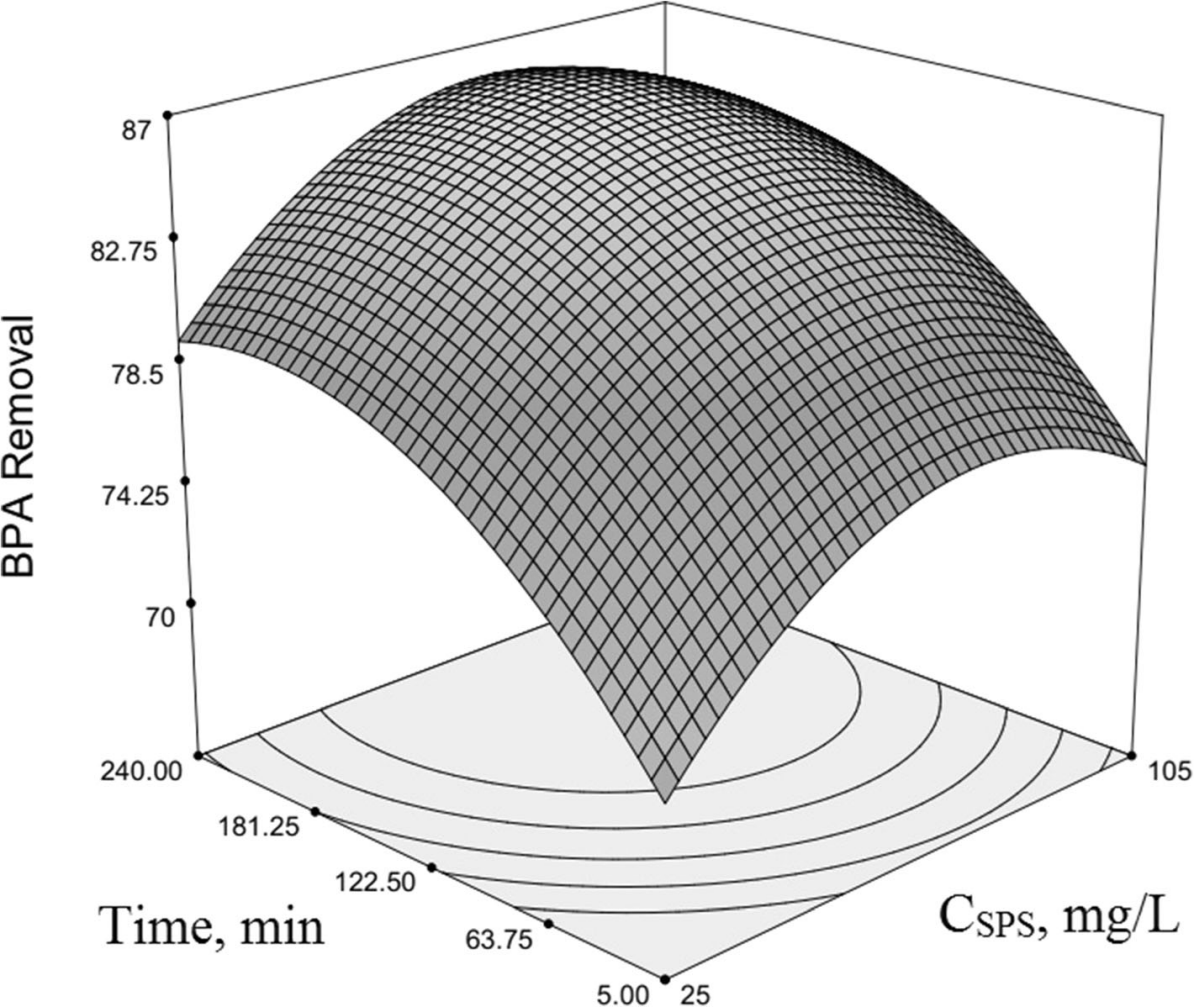
Second-order response surface plot in the BPA removal for the UV/SPS/HP/Cu system. Dependence of y on the SPS and time. (HP = 10 mg/L, pH = 7, Cu^2+^ = 17.5 mg/L, BPA = 22.5 mg/L)

**Fig. 5:**
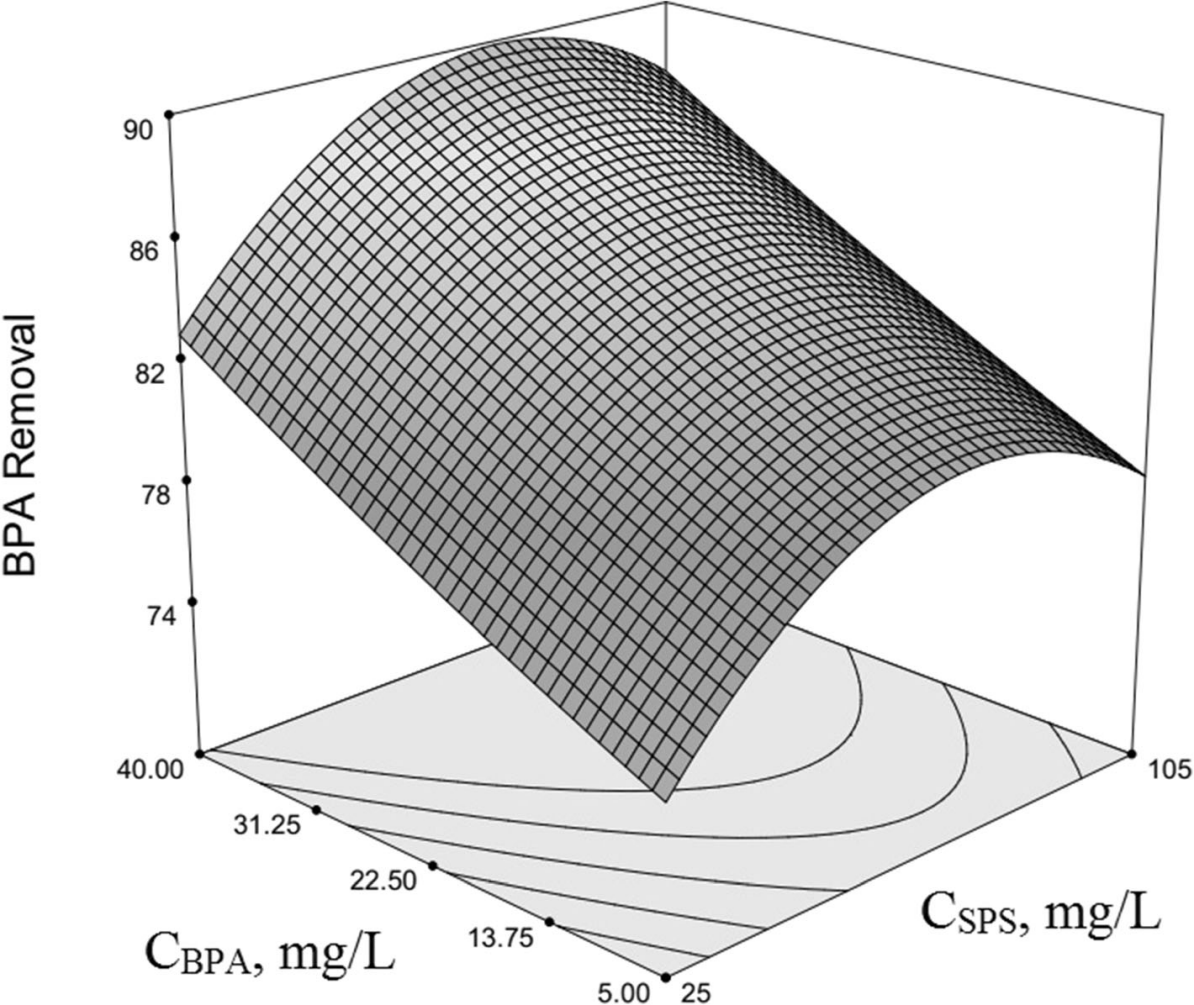
Second-order response surface plot in the BPA removal for the UV/SPS/HP/Cu system. Dependence of y on the SPS and initial BPA concentrations. (HP = 10 mg/L, pH = 7, Cu^2+^ = 17.5 mg/L, Time = 122.5 min)

The use of three-dimensional plots of the regression model is highly recommended for the graphical interpretation of the interactions [54–56]. Variables that giving quadratic and interaction terms with the largest absolute coefficients in the fitted model, were chosen for the axes of the response surface plots to account for the curvature of the surfaces. SPS and HP concentration were selected for the RSM plots of BPA removal, while other factors (*x*_*3*_ – *x*_*6*_) were kept at the central levels (Fig. 1), and also in the next figures (Figs. 2, 3, 4 and 5) SPS concentration and pH, SPS and Cu^2+^ concentrations, SPS concentration and reaction time, and SPS and initial BPA concentrations were selected respectively as variables while four other factors were constant as cantered levels at the same time. Resulted interaction indicates that the effect created by variation of the SPS concentration, as a regressor variable depends on the value of HP as the another regressor variable (Fig. 2), and similarly it depends on the levels of the other variable as regressor (*x*_*3*_ – *x*_*6*_) and also all of the factors have interactions with each other based on the model. In the obtained model, *x*_*6*_ is the principle regressor variable influencing the responses (Highest coefficients, β_1_s) whereas this variable had an interaction with *x*_*1*_ equal to + 0.13 (β16); and according to Figs. 1, 2, 3, 4 and 5, SPS concentration (*x*_*1*_) has interactions with *x*_*2*_, *x*_*3*_, *x*_*4*_ and *x*_*5*_ equal to −0.12, −0.04, −0.083 and −0.2 respectively. The plots show a relative high degree of curvy of 3-dimensional surfaces (Upwards or downwards depend on interaction type and degree).

The removal of BPA increased when SPS concentration raised to its centred level (65 mg/L, obtained as the ratio to BPA), and increasing of it towards +1 level (105 mg/L) results in remaining constant and slightly reduced in BPA removal; simultaneously with increasing HP concentration, BPA removal starts to increase and about in centre level (10 mg/L) it is reached to maximum level, and then starts to decrease with increasing HP concentration to the high level (15 mg/L) (Fig. 1). In this system, in addition of UV irradiation, that had a constant intensity (30 W UV lamp), other chemicals as variables have been used as compilation and integrated. The reason of efficiency reducing can be attributed to being constant of BPA mole number as reactant that will react with increased PS concentration and therefore sulphate radicals for degradation; this means that possibility of contact between radicals and BPA will not increase so much and increasing of SPS concentration will be result in slightly increase in reaction rate in the first minutes of process, but by continuing of reaction and reduction in BPA concentration in the reactor, reaction rate again reduce greatly because of reduced numbers of BPA moles; results of Yang (2009), Olmez-Hanci (2013) and Liang (2013) confirmed also obtained results in this work.

However, the presence of excess HP moles as SPS activator and also responsible agent for HO^•^ radical, can be play as a radical scavenger for SO_4_^-•^ and again reconvert it to SO_4_^2−^ ion that cause reduced in reaction rate and then BPA removal. An excessively high initial PS concentration can lead to the generation of a higher quantity of SO_4_^-•^ that may inhibit BPA oxidation according to the following equation [57–59]:

6 $$ \mathrm{S}{{\mathrm{O}}_4}^{\hbox{-} \bullet } + \mathrm{S}{{\mathrm{O}}_4}^{\hbox{-} \bullet}\to\ {\mathrm{S}}_2{{\mathrm{O}}_8}^{2\hbox{-} } $$

7 $$ \mathrm{S}{{\mathrm{O}}_4}^{\hbox{-} \bullet } + \mathrm{H}{\mathrm{O}}^{\bullet}\to\ \mathrm{S}{{\mathrm{O}}_4}^{2\hbox{-} } + \mathrm{O}{\mathrm{H}}^{\hbox{-} } $$

Reviews of Fig. 2 illustrates that BPA oxidation with this system completely depend on pH; and SPS concentration and pH has interaction with each other, which their interaction is reflected in model with coefficient equal 0.04. In addition to the SPS behaviour described in the previous section, pH also plays an important role in the process, and therefore in the neutral pH, the system has the minimum efficiency and at the acidic and basic pH is more efficient, so that pH =11 had been the highest efficiency.

It has already been demonstrated that in acidic condition SO_4_^−•^ are the dominant radical and in alkaline conditions can induce the mechanism of SO_4_^−•^ interconversion to HO^•^ in the PS activation system and SO_4_^−•^ can undergo reactions with hydroxide ions in accordance with Eq. (8) to generate hydroxyl radicals, in addition, SO_4_^−•^ may react with water at all pHs to produce HO^•^, according to the following equations. However, Norman et al. reported that the reaction rate constant of Eq. (9) is low in comparison to those of SO_4_^−•^ reactions with organic compounds [57, 60–62]:

8 $$ \mathrm{In}\ \mathrm{alkaline}\ \mathrm{range}\ \mathrm{of}\ \mathrm{p}\mathrm{H}:\ \mathrm{S}{{\mathrm{O}}_4}^{\hbox{-} \bullet } + \mathrm{O}{\mathrm{H}}^{\hbox{-}}\to\ \mathrm{S}{{\mathrm{O}}_4}^{2\hbox{-} } + \mathrm{H}{\mathrm{O}}^{\bullet }\ \mathrm{k} = 6.5 \pm 1 \times 1{0}^7{\mathrm{M}}^{\hbox{-} 1}{\mathrm{s}}^{\hbox{-} 1} $$

9 $$ \mathrm{In}\ \mathrm{all}\ \mathrm{ranges}\ \mathrm{of}\ \mathrm{p}\mathrm{H}:\ \mathrm{S}{{\mathrm{O}}_4}^{\hbox{-} \bullet } + {\mathrm{H}}_2\mathrm{O}\ \to\ \mathrm{S}{{\mathrm{O}}_4}^{2\hbox{-} } + \mathrm{H}{\mathrm{O}}^{\bullet } + {\mathrm{H}}^{+}\kern0.5em {k_{\mathrm{H}}}_{2\mathrm{O}} < 2 \times 1{0}^{\hbox{-} 3}{\mathrm{s}}^{\hbox{-} 1} $$

It should be mentioned the hydroxyl radical has a higher redox potential and a less-selective reactivity to contaminant degradation. Therefore, the formation of HO^•^ potentially increases the degradation rate of the BPA and intermediate compounds in alkaline pH; as well as SO_4_^−•^ and HO^•^ react with organic compounds mainly by three main mechanisms: hydrogen abstraction, hydrogen addition, and electron transfer [61], and generally SO_4_^−•^ is more likely to participate in electron transfer reactions than HO^•^, which is more likely to participate in hydrogen abstraction or addition reactions [63, 64]; hence reactivity of HO^•^ is less selective than SO_4_^−•^. Furthermore, Liang and et al., using a chemical probe method on a thermally activated persulfate oxidation system, identified dominant radical species in different pH conditions: at basic pH hydroxyl radical is dominant, in the neutral pH both SO_4_^−•^ and HO^•^ are present, and in the acidic pH dominant specie is SO_4_^−•^ [65]. The results of this work about pH effect were confirmed by the results of Ya-Ting et al., on the ultraviolet activated persulfate oxidation of phenol, and results of Sung-Hwan et al., on the oxidation of BPA with UV/HP and UV/SPS [44, 61].

Copper cation (Cu^2+^) interaction with PS has been investigated in Fig. 3 in accordance of obtained model. As seen in the model the BPA removal increased as Cu^2+^ concentration increased from −1 level to its central level (17.5 mg/L, obtained as the molar ratio to BPA), and then starts to decrease when Cu^2+^ is increasing towards +1 level (30 mg/L); simultaneously PS has almost the similar behaviour in the previous steps.

Usually to enhance the oxidation of contaminants, PS oxidation is conducted under different methods of activation, and transition metals, especially divalent metals (Cu^2+^, Fe^2+^, and …) which are commonly present and are important in soil and groundwater systems, also act as electron donors to catalyse the decomposition of SPS through a one-electron transfer reaction analogous to the Fenton initiation reaction, and according to the following equation activation of SPS result in sulphate radical that applied for degradation of BPA. However, excessive Cu^2+^ can significantly scavenge SO_4_^−•^ and inhibit radical oxidation of target pollutant, BPA, via Eq. (11) [45, 66, 67].

10 $$ {\mathrm{S}}_2{{\mathrm{O}}_8}^{2\hbox{-} } + \mathrm{C}{\mathrm{u}}^{2+}\to \mathrm{S}{{\mathrm{O}}_4}^{\hbox{-} \bullet } + \mathrm{S}{{\mathrm{O}}_4}^{2\hbox{-} } + \mathrm{C}{\mathrm{u}}^{3+} $$

11 $$ \mathrm{C}{\mathrm{u}}^{2+}+\mathrm{S}{{\mathrm{O}}_4}^{\hbox{-} \bullet}\to \mathrm{C}{\mathrm{u}}^{3+} + \mathrm{S}{{\mathrm{O}}_4}^{2\hbox{-} } $$

Results of Krzysztof Kuśmierek et al., about oxidative degradation of 2-chloophenol by PS showed that synergistic activation of PS by heat and Cu^2+^ was more effective than heat and alkali activation and excess Cu^2+^ resulted in a relative decrease in removal efficiency [68]; Results of this work compatible with the present work. And in another work Liu, C. S., et al. studied oxidative degradation of propachlor by ferrous and copper ion activated persulfate. Results showed the activation of PS by Fe^2+^ ions resulted in rapid degradation of propachlor in the early stage, but was accompanied by a dramatic decrease in efficiency due to the rapid depletion of Fe^2+^ by the sulphate radicals generated, but the Cu^2+^ activated PS had a longer lasting degradation effect and a proportionally greater degradation enhancement at elevated Cu^2+^ concentrations [45].

Figure 4 indicates reaction time (min) interaction with SPS concentration (mg/L) on the BPA degradation rate in the range of 240 min based on the obtained model. Other effective factors were kept constant as cantered levels. As obviously seen in the graph, with the progress of reaction and increasing of time, BPA degradation and its removal was enhanced noticeably, while SPS concentration ranged from −1 to +1 levels as coded values, and in all of SPS levels, BPA has been increased with time reaction. So that at BPA concentrations of 22.5 mg/L, with increasing SPS concentration from −1 to +1 levels, the BPA removal rate first increased and then remained constant and finally declined slightly. In addition, gradient of the graph was up and then slightly was decreased. This is due to consumption and reduce of reactant mole numbers over time, and thereby reducing the probability of collisions between them [58]. The other studies have been similar results on the effect of reaction time [47, 67, 69–72].

Figure 5 shows the second-order response surface plot for the BPA removal by UV/SPS/HP/Cu system that indicates the interaction influence of BPA initial concentration (mg/L) and SPS concentration (mg/L) on the BPA degradation rate at the centred level of other factors. Figure 5 demonstrate that, with increasing SPS and BPA initial concentrations, the BPA degradation rate has considerably improved. Although PS has continued its quadratic effect, BPA initial concentration almost has a linear effect, which means that any increase in its level could lead to increase in degradation rate and removal efficiency. These results suggest that with increasing of BPA concentration under applied process with given conditions, reactants including sulphate and hydroxyl radicals could have more opportunities for encounter with the target pollutant, BPA, that can be lead to increasing the degradation rate and removal efficiency of BPA. In addition, using the increased level of PS can lead to more radicals, and synchronization of this phenomenon along with increased the initial level of BPA, can cause to more faster degradation of BPA [73, 74]. The obtained results of this work are confirmed with similar study’s results by application of the PS and other AOPs processes for BPA removal from various fields of environment [57, 61, 71, 75, 76].

For optimization of BPA removal by changing selected factors and with choosing criteria goal as “in range” for all six variables as affecting factors and “maximize” as goal for BPA removal using model, the optimum values of operation parameters were obtained as: initial SPS concentration (69.57 mg/L), initial HP concentration (9.85 mg/L), initial pH (10.91), initial Cu^2+^ concentration (20.76 mg/L), reaction time (140.38 min), and BPA initial concentration (39.33 mg/L), respectively, leading to 99.99 % of BPA removal.

According to the kinetic study carried out in optimal condition, BPA degradation using this system follow the first-order kinetic model and reaction rate was a function of time and initial concentration BPA. Reaction rate constant (K) was calculated in range 0.0073–0.0433 min^−1^ for initial BPA concentration in range 5–40 mg/L. Also, study of system as separate process (for example: UV alone) showed that using system as combined form, have synergistic effect than separate processess (Table 4) [77, 78]. Quenching studies with methanol (MA) and tert-butyl alcohol (TBA) were performed to identify the primary radical species formed in the system, showed that K reduced from 0.049 min^−1^ to 0.0116 min^−1^ and 0.0176 min^−1^ for MA and TBA, respectively. These results demonstrate that both SO_4_^−•^ and HO^•^ can degrade BPA, but SO_4_^−•^ plays the dominant role. Results of Yang and Hanci’s study on removal of Azo dye acid Orange 7 (AO7) and BPA matches with results of this work [47, 57].

**Table 4:** Synergistic effect of the UV/SPS/HP/Cu in different arrangements (Optimal Condition)

No.	Process	K (min^−1^)					Synergistic effect	
		UV	SPS	HP	Cu	Combined	S	S’
1	Distinct process	×	×	-	-	-	-	-
2	UV/SPS	×	-	×	-	0.0073	1.7	41.9
3	UV/HP	×	-	-	×	0.006	1.6	37.5
4	UV/Cu	-	×	×	-	0.0034	1.3	22.7
5	SPS/HP	×	-	-	×	0.005	1.7	41.1
6	SPS/Cu	-	×	-	×	0.004	1.6	36.9
7	HP/Cu	×	×	×	-	0.0036	1.9	47.2
8	UV/SPS/HP	×	×	-	×	0.018	3.3	70
9	UV/SPS/Cu	×	-	×	×	0.011	2.4	58.5
10	UV/HP/Cu	-	×	×	×	0.0096	2.5	60
11	SPS/HP/Cu	×	×	×	×	0.0075	2.4	58.5
12	UV/SPS/HP/Cu	×	×	-	-	0.043	7.8	87.14

$$ *S=\frac{k\left(A+B\right)}{k(A)+k(B)},\;S\mathit{\hbox{'}}=\frac{k\left(A+B\right)-\left[k(A)+k(B)\right]}{k(A)+k(B)}\times 100 $$ [78, 79]

Finally, to evaluation of system performance in mineralisation of BPA in optimal condition, TOC analysis was performed. Results showed that process was able to remove up to 70 % of TOC in 180 min of reaction time. This means that mineralisation of BPA was done.

### Toxicity study and GC-MS analysis

**Fig. 6:**
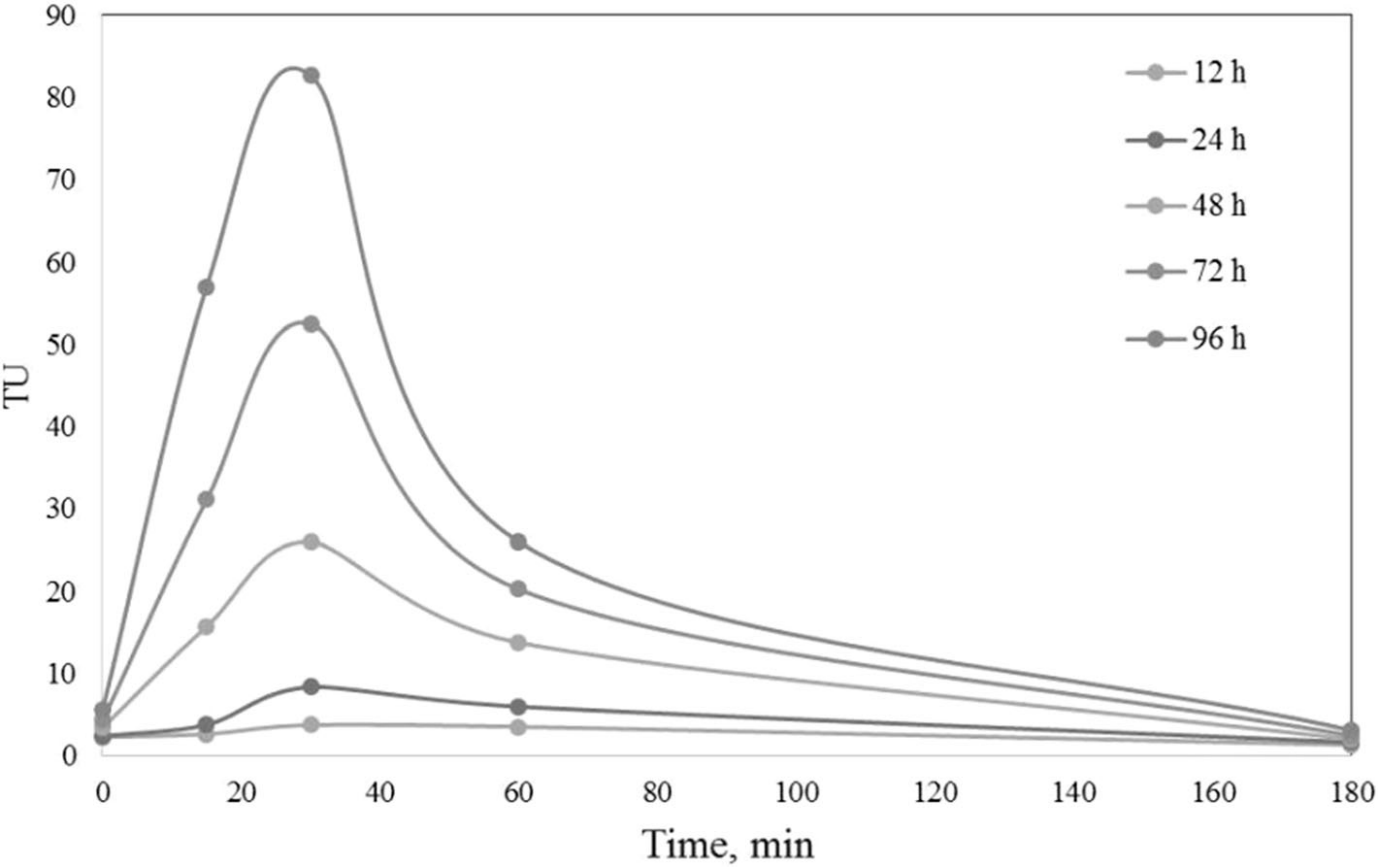
Toxicity of BPA and its intermediate products during the reaction, based as acute toxicity unite (TUa) in the optimum conditions. [BPA]_i_ = 0.219 mmol

**Fig. 7:**
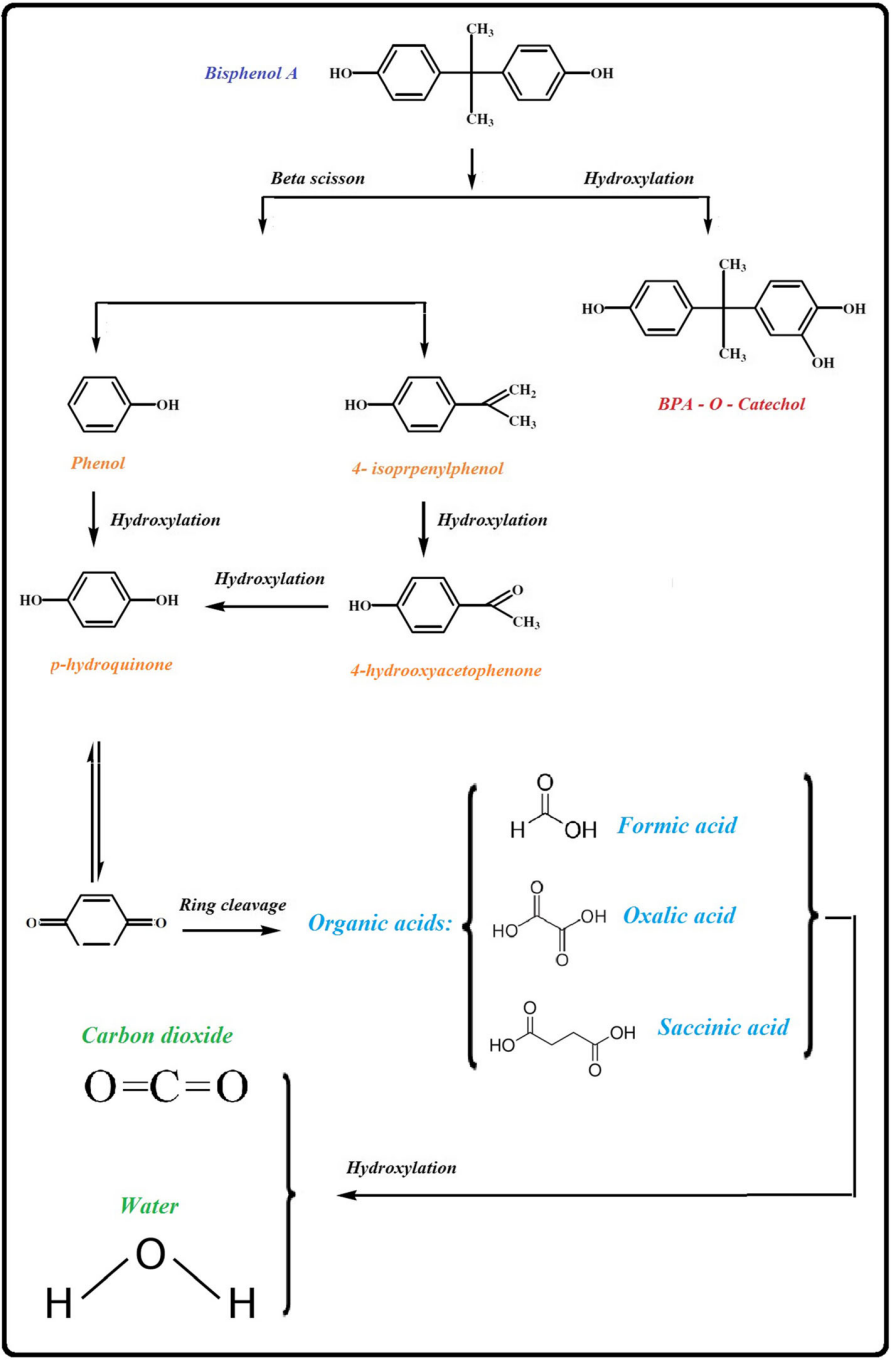
Possible pathways of BPA degradation induced by applied UV/SPS/HP/Cu system

Results of bioassay for start point, and for times 15, 30, 60 and 180 min after beginning of reaction time is presented in Fig. 6. As shown in mentioned figure toxicity of BPA was increased during the reaction and then after 30 min starts to reduce and finally at the end of process reached to a minimum (24 h. toxic was increase from 2.35 TU to 8.34 TU in 30 min and then reduced to 1.58 TU at the end of reaction). This can be attributed to the production of intermediate products (aromatic and aliphatic as shown in Fig. 7) during the reaction, which probably are more toxic than BPA [66, 79]. Dehghani and et al. also was obtained similar results with ultrasonic and hydrogen peroxide processes and reach to < 2 TU [80]. Results of Tugba Olmez-Hanci’s study by hot PS process with bioluminescence inhibitor are compatible with present study and has the same trend in toxicity along the process [57].

Several mechanisms have been proposed for better understanding the BPA degradation pathways by HO^•^-based AOPs [81, 82]. However, so far, there are very few reports related to the BPA oxidation pathway by SO_4_^−•^-based AOPs process, especially UV/SPS/HP/Cu process. It must be considered that the degradation of organic pollutants is often done by the formation of some intermediates that can potentially impose toxic effect in the environment.

So, oxidation products formed during the UV/SPS/HP/Cu treatment of BPA were analysed by GC-MS after 0, 15, 30, 60 and 180 min treatment. Based on the obtained mass spectra, the evolution of several oxidation products was evident. Some of identified products include BPA catechol, 3-Hydroxy-4-methyl-benzoic acid, 2,3-dimethyl benzoic acid, 4-hydroxyacetophenone, 4-isopropenylphenol, Benzaldehyde, Succinic acid, p-hydroquinone, Phenol, Ethylene glycol monoformate, and numbers of unknown compounds that some of them heavier and some of them had lighter molar weight than BPA. The finding of similar studies using various AOPs processes, confirm presence of some mentioned compounds and some other similar compounds in treated effluents due to degradation of BPA [42, 49, 57, 82, 83]. Continuing of process results in more reduction in these compound’s concentration and therefor complete mineralization of BPA after 180 min (Results are not shown). These trend of BPA degradation and production of intermediates during the process explains changes in effluent toxicity during the treatment; and it has been postulated that the intermediates formed during the initial stages of BPA oxidation are more toxic than BPA itself and according to Zazo et al., the subsequent decrease in the toxicity corresponds to the disappearance of aromatic intermediates giving rise to organic acids of relatively low toxicity [57, 84]. Considering that SO_4_^−•^ and HO^•^ react with organic compounds by hydrogen abstraction, hydrogen addition, and electron transfer mechanisms [61], and as discussed in the last sections, generally SO_4_^−•^ is more likely to participate in electron transfer reactions along with HO^•^ that tends to two other mechanism, and according to the produced intermediates mentioned above, possible degradation pathway as simplified can be assumed as Fig. 7.

Finally, to evaluate the remaining parameters in the effluents including SPS, HP and Cu^2+^ in optimum conditions and after completing the process, a sample from effluent were analysed based on standard methods. Aaccording to the results, remaining concentrations of PS, HP and Cu^2+^ was respectively 32.5 mg/L, 4.9 mg/L and 8.75 mg/L. Considering that probably after treatment, effluents enter to the receiving waters, and for continuing process and degradation and mineralization of intermediates produced in the process, the mentioned remaining values will be useful, and will also meet effluent standards.

### Evaluation of system performance on real effluent

Due to presence of BPA in real effluents with different conditions that distinguish it from synthetic wastewater, obtained optimal conditions were applied for treating a sample from effluent of plastic industry. Initial specifications of mentioned sample were presented in Table 5. Results showed that BPA reduced to 42 μg/L (about 77 % removal) and TOC was removed about 51 %. These explain that UV/SPS/HP/Cu system is proper process in BPA removal and it can be used for treating of the variety effluents contain BPA, but reason of low-performance of system for real conditions can be attributed to the presence of other factors as inhibitors in the content of wastewater. So, if this system applied for real conditions, it is recommended that further experiments shall be done for achieve optimum condition of removal and mineralization of BPA.

**Table 5:** The physicochemical characteristics of used real effluent (Plastic industry, polycarbonate)

Parameter	Concentration, mg/L
pH	6.5
Cu	3.5
Hardness	385
Chloride	340
TOC	1760
Bicharbonate	280
BPA	145 μg/L

## Conclusion

The purpose of this study was to investigate degradation and mineralisation of BPA in the aqueous solutions as well as optimization BPA removal from aqueous solution using UV/SPS/HP/Cu process. Based on our knowledge, this work is carried out for the first time using a statistical experimental design for optimization of BPA removal by combined UV/SPS/HP/Cu activated persulfate process. A six-variable, three-level CCD in combination with response surface modelling and quadratic polynomial were used to determine of the effects of initial SPS concentration (25–105 mg/L), initial HP concentration (5–15 mg/L), initial pH (3–11), initial Cu^2+^ concentration (5–30 mg/L), reaction time (5–240 min), and BPA initial concentration (10–40 mg/L) on the degradation of BPA as the response variable. Graphical response surface and contour plot were applied for the optimization of reaction condition.

The results of analysis of the response surfaces revealed that the six independent variables and their interactions have been significant effects on BPA degradation rate. The BPA initial concentration showed the highest effect, followed by reaction time, pH, SPS concentration, HP concentration and Cu^2+^ concentration.

With selecting criteria goal as “in range” for all six variables and “maximize” as goal for BPA removal, the optimum values of operation parameters were obtained as: initial SPS concentration (69.57 mg/L), initial HP concentration (9.85 mg/L), initial pH (10.91), initial Cu^2+^ concentration (20.76 mg/L), reaction time (140.38 min), and BPA initial concentration (39.33 mg/L), respectively, leading to 99.99 % of BPA removal. As well as, the ANOVA results indicated relatively high coefficient of the determination values (R^2^ = 0.9622 and Adj-R^2^ = 0.9447). The assessment of the model validation and adequacy also indicated a satisfactory goodness-of-fit obtained between the actual and predictive values by the model.

TOC analysis and bioassay with D. Magna revealed that BPA has been mineralised, toxicity of effluent reduced, and capable to discharge to the environment. Finally, the results of this study showed that UV/SPS/HP/Cu system is a new and efficient method in removal and mineralisation of BPA from aqueous solutions; moreover, CCD from the response surface methodology was an efficient statistical technique for the optimization of BPA removal from aqueous solutions using UV/SPS/HP/Cu process.

## Abbreviations

μg/L: microgram per litre
BPA: Bisphenol A
C.V.: Coefficient of variance
CCD: Central composite design
CS: Calibration standard
EPA: Environmental Protection Agency
FDA: Food and Drug Administration
HO^•^: Hydroxyl Radical
HPLC: High-performance liquid chromatography
mg/L: milligram per litre
NF: Nano filtration
PNEC: Predicted no effect concentration
PS: Persulfate
QC: Quality control
RO: Reverse osmosis
RSM: Response surface methodology
SO_4_^−•^: Sulfate Radical
SPS: Sodium persulfate
TOC: Total organic carbon
UV: Ultraviolet
WWTPs: Wastewater treatment plants

## Footnotes

1 Predicted no effect concentration
